# Dr. Jack’s Views on Implantation

**Published:** 1896-11

**Authors:** 


### Dr. Jack's Views on Implantation.

It is unfortunate that in too many instances planted teeth, which have become fixed and useful organs, have, after several years, from causes not entirely clear, yielded to absorption and have become useless. The termination of these cases seem to come on with suddenness and without warning of the resorptive process. The cause of this result we have to look for in a depressed trophic state of the environment, in which condition it is not unreasonable to expect tissue of repair to be the first to yield. In any defective condition of the nutrition of a part this result is in consonance with well-fixed principles.

We have also to consider that the alveolar process is a provisional structure, and that its tendency is, on the occurrence of irritation or of impaired nutrition or by static conditions, to yield to resorption. The fact that it is provisional would impress on it the tendency to this structural change.

Notwithstanding the prognosis of planted teeth is at present precarious, the promise of useful operations is so large, when selection is made of subjects in sound health and without cachetic taints, that, where plantation is required for the good of the patient, it should be performed. The probabilities are that greater experience will lead to improved methods and more certain results.—*Cosmos.*

